# Should Kidney Transplantation be Offered to Patients with Body Mass Index >40?: CON

**DOI:** 10.34067/KID.0000000711

**Published:** 2025-08-08

**Authors:** Tomoki Tsukahara, Rita L. McGill

**Affiliations:** Section of Nephrology, Department of Medicine, University of Chicago, Chicago, Illinois

**Keywords:** kidney transplantation, obesity

Obesity is increasing rapidly around the globe, and kidney transplant candidates are no exception to this trend. Metabolic syndrome and diabetes, the downstream risks of obesity, have helped drive the explosive growth of ESKD in this country. United Network for Organ Sharing data show that more than one third of kidney recipients had a body mass index (BMI) >30 kg/m^2^ in 2022, despite Kidney Disease Improving Global Outcomes guidelines in 2020 that describe obesity as a relative contraindication to kidney transplantation.^1^ Many transplant centers have adopted individual BMI thresholds, usually 35–40 kg/m^2^.

Increasing acceptance of obese candidates occurred after the seminal article by Gill *et al.* showing a survival benefit for most obese kidney recipients, compared with their counterparts on the kidney transplant waitlist.^2^ Over the subsequent 10 years, six independent large meta-analyses have nonetheless shown that obese recipients face substantially increased mortality, delayed graft function (DGF), graft failure, acute rejection, and new-onset diabetes and that these risks have not attenuated with advances in transplantation methods over time (Table 1). Some of these studies indicate that risks of obesity are dose dependent, with patients with class 3 obesity (BMI >40 kg/m^2^) incurring the highest risks (Table 1). In prior work by our group, mate-kidney analyses demonstrated that both death-censored graft failure and patient mortality increased almost linearly with increasing BMI, without any obvious threshold values. Although recipients with BMI >35 kg/m^2^ did as well as recipients with BMI 30–35 kg/m^2^, both groups had inferior outcomes compared with nonobese and overweight patients.^3^ Compared with patients with BMI=25 kg/m^2^, those with BMI >40 kg/m^2^ had a 24% increased risk of death and 26% increased risk of death-censored graft failure (*P* < 0.005 for both).^3^

**Table 1:** Synopsis of meta-analyses concerning transplantation outcomes among obese patients

Author, Year	Reference BMI Level	Obesity BMI Level	Statistic	DGF	Death-Censored Graft Failure	Graft Failure	Mortality	Acute Rejection	New-Onset Diabetes
Ahmadi *et al.*,^11^ 2014	<30	30–35	HR	—	1.10 (1.05–1.16)	1.13 (1.09–1.17)	1.16 (1.11–1.21)	—	—
	<30	35–40	HR	—	1.32 (1.23–1.41)	1.25 (1.18–1.31)	1.22 (1.14–1.30)	—	—
	<30	>40	HR	—	1.35 (1.21–1.50)	1.34 (1.23–1.45)	1.37 (1.24–1.52)	—	—
Nicoletto *et al.*,^12^ 2014	<30	≥30	RR	1.41 (1.26–1.57)	—	1.31 (1.11–1.51)	—	0.95 (0.82–1.11)	—
LaFranca *et al.*,^13^ 2015	<30	≥30	RR	1.52 (1.35–1.72)	—	1.03 (1.01–1.04)	1.52 (1.14–2.03)	1.17 (1.01–1.37)	2.24 (1.01–1.37)
Hill *et al.*,^14^ 2015	<30	≥30	OR	1.68 (1.39–2.03)	—	—	—	—	—
	<30	≥30	HR	—	1.06 (1.01–1.12)	—	1.24 (0.90–1.70)	—	—
Sood *et al.*,^15^ 2016	<30	≥30	OR	1.81 (1.51–2.13)	—	—	—	—	—
	<30	≥30	HR	—	1.54 (1.38–1.68)	—	1.19 (1.10–1.31)	1.51 (1.24–1.78)	1.01 (0.98–1.07)
Yin *et al.*,^16^ 2022	22	32	RR	1.38 (1.35–1.42)	—	—	—	—	2.02 (1.27–3.33)
	22	36	RR	1.68 (1.63–1.74)	—	—	—	—	—
	22	40	RR	2.05 (1.96–2.13)	—	—	—	—	—
	22	32	HR	—	—	1.13 (1.04–1.24)	1.32 (1.00–1.76)	1.08 (1.01–1.16)	—
	22	36	HR	—	—	1.37 (1.21–1.54)	1.89 (1.16–3.08)	1.17 (1.12–1.22)	—
	22	40	HR	—	—	1.69 (1.43–2.00)	2.63 (1.38–5.02)	1.28 (1.19–1.37)	—

BMI, body mass index; DGF, delayed graft function; HR, hazard ratio; OR odds ratio; RR, risk ratio.

Obesity affects transplant outcomes by many mechanisms. Visceral adipose tissue releases cytokines, inducing a proinflammatory/oxidative state, with recruitment of macrophages and monocytes.^4,5^ The renin-angiotensin-aldosterone system is activated, exposing patients to the deleterious effects of unremitting angiotensin II and aldosterone, which creates a profibrotic milieu. There is also endothelial dysfunction, with reduced nitric oxide, and a prothrombotic state mediated by excess plasminogen activator.^5^ Perivascular fat inflammation and fibrosis lead to vascular stiffness.^4^ Taken together, these factors cause deterioration in the glomerular, interstitial, and vascular compartments of the kidney, whether a native organ or allograft kidney. In the setting of visceral obesity, there is arterial sclerosis, expansion of glomerular and interstitial matrix, and chronic inflammation. These changes result in glomerular hyperfiltration, hypertension, and albuminuria.^4,5^ In this setting, increased hyperlipidemia, hypertension, and insulin resistance leading to type 2 diabetes also contribute to increased mortality.

The perioperative risks of any surgery are much higher for patients with obesity. Obesity complicates ventilatory management during and after surgery.^6^ Mechanical pressure exerted by excess weight can compromise surgical incisions, leading to dehiscence and hernia. After surgery, obese patients are at increased risk of reduced mobility, hypoventilation, and venous thromboembolism.^6,7^ Among kidney transplant recipients, obesity is associated with increased DGF, death-censored graft failure, acute allograft rejection, wound infection, wound dehiscence, increased hospital length of stay, and risk of readmission (Table 1). Operative times are longer, even when robotic approaches are used, and the increased warm ischemia time would intuitively predispose to DGF and anesthesia complications. Once out of surgery, the obese patient continues to face disadvantages. Reduced blood supply in adipose tissue predisposes to slow wound healing and greater risk of surgical site infections.^6,7^ The optimal dosing of immunosuppressive medications in obese patients poses further complexity. Adipose tissue does not contribute predictably to the volume of distribution for calcineurin inhibitors, so the conventional practice of dosing per kilogram body weight often results in inconsistent drug levels, with renal vasoconstriction when levels are high and an increased risk of acute rejection when drug levels are low. Data also show that obese patients have a higher incidence of rejection with the use of belatacept.^8^

The risks of severe obesity may be reduced by weight loss interventions before transplantation, although this is not uniformly accepted. However, even with resource-intensive support systems, diet and exercise programs are often ineffective, and weight lost is rarely maintained. Surgical approaches to weight loss, such as gastric sleeve and Roux-en-Y gastric bypass, have been examined in carefully selected obese transplant candidates. In a meta-analysis of 2317 obese kidney transplant candidates, bariatric surgery was shown to be an effective method to reduce BMI by an average of approximately 11 kg/m^2^, with a combined surgical mortality of 4%.^9^ Most reports of bariatric surgery in transplant candidates reveal substantial patient dropout, suggesting that patients seeking organ transplants hesitate to undergo this surgery, even when highly motivated to avoid dialysis. There is growing interest in glucagon-like peptide-1 receptor agonists and glucose-dependent insulinotropic polypeptide receptor antagonists, which have been shown to promote weight loss in patients with and without diabetes. Glucagon-like peptide-1 receptor agonists and glucose-dependent insulinotropic polypeptide receptor antagonists have only recently begun to be used in patients with advanced kidney disease and ESKD. Vanek *et al.* reported 12 weeks of subcutaneous semaglutide therapy in 13 kidney transplant candidates receiving dialysis who had been denied waitlisting because of obesity. Although three candidates were re-evaluated for transplant waitlisting, their median weight loss was only 4.6 (interquartile range, 2.0–9.7) kg at week 12.^10^

Despite efforts to promote kidney transplantation, the supply of donor organs has never been equal to the ever-growing waiting list. In a resource-limited situation, we owe it to our patients to seek the best distribution of kidneys, optimizing the quality and quantity of benefits realized by recipients. Clinicians are therefore obligated to pay attention to the ethical balance between benefits and harms. Increased mortality, morbidity, and graft failure among kidney recipients with obesity are disturbing and should not be ignored. While the overall survival of obese patients is superior after transplantation, individual patients may suffer greatly and have shorter survival than they might have realized while receiving dialysis. Graft loss in this setting is a tragedy not only for the obese recipient, but also for other important stakeholders. Many patients die awaiting transplant, having never had the opportunity to receive a kidney. We must also recognize our obligations to living donors who contribute their kidneys and the families of deceased donors, whose generous decision often provides some meaning to the pain of bereavement. We need to consider all of these individuals and work to optimize the value of their gifts.

Consideration of all of this evidence leads us back to the wisdom of the Kidney Disease Improving Global Outcomes guidelines, which is to approach the kidney transplant candidate with class 3 obesity with great care and caution, considering the increased risks of postoperative complications and the benefits of kidney transplantation.^1^

## References

[1] Chadban SJ Ahn C Axelrod DA, . Kidney disease: improving global outcomes (KDIGO) kidney transplant candidate work group. KDIGO clinical practice guideline on the evaluation and management of candidates for kidney transplantation. Transplantation. 2020;104:S1–S103. doi:10.1097/TP.000000000000316

[2] Gill JS Lan J Dong J, . The survival benefit of kidney transplantation in obese patients. Am J Transplant. 2013;13(8):2083–2090. doi:10.1111/ajt.12331 23890325

[3] Sureshkumar KK Chopra B Josephson MA Shah PB McGill RL. Recipient obesity and kidney transplant outcomes: a mate-kidney analysis. Am J Kidney Dis. 2021;78(4):501–510.e1. doi:10.1053/j.ajkd.2021.02.332 33872689

[4] Whaley-Connell A Sowers JR. Obesity and kidney disease: from population to basic science and the search for new therapeutic targets. Kidney Int. 2017;92(2):313–323. doi:10.1016/j.kint.2016.12.034 28341271

[5] Glicklich D Mustafa MR. Obesity in kidney transplantation. Impact on transplant candidates, recipients, and donors. Cardiol Rev. 2019;27(2):63–72. doi:10.1097/CRD.0000000000000216 29870421

[6] Kaw R Dupuy-McCauley K Wong J. Screening and perioperative management of obesity hypoventilation syndrome. J Clin Med. 2024;13(17):5000. doi:10.3390/jcm13175000 39274213 PMC11396152

[7] Stumpf MAM Mancini MC. Challenges in the care and treatment of patients with extreme obesity. Arch Endocrinol Metab. 2024;68(68):e230335. doi:10.20945/2359-4292-2023-0335 39420906 PMC11326745

[8] Lange NW King K Husain SA, . Obesity is associated with a higher incidence of rejection in patients on belatacept: a pooled analysis from the BENEFIT/BENEFIT-EXT clinical trials. Am J Transplant. 2024;24(6):1027–1034. doi:10.1016/j.ajt.2024.02.015 38387620 PMC11930353

[9] Fernando S Varma J Dengu F Menon V Malik S O’Callaghan J. Bariatric surgery improves access to renal transplantation and is safe in renal failure as well as after transplantation. A systematic review and meta-analysis. Transplant Rev (Orlando). 2023;37(3):100777. doi:10.1016/j.trre.2023.100777 37459746

[10] Vanek L Kurnikowski A Krenn S Mussnig S Hecking M. Semaglutide in patients with kidney failure and obesity undergoing dialysis and wishing to be transplanted: a prospective, observational, open-label study. Diabetes Obes Metab. 2024;26(12):5931–5941. doi:10.1111/dom.15967 39375862

[11] Ahmadi S-F Zahmatkesh G Streja E, . Body mass index and mortality in kidney transplant recipients: a systematic review and meta-analysis. Am J Nephrol. 2014;40(4):315–24. doi:10.1159/000367812 25341624 PMC4319181

[12] Nicoletto BB Fonseca NKO Manfro RC Gonçalves LFS Leitão CB Souza GC. Effects of obesity on kidney transplantation outcomes: a systematic review and meta-analysis. Transplantation. 2014;98(2):167–176. doi:10.1097/TP.0000000000000028 24911038

[13] LaFranca JA IJermans JNM Betjes MGH Dor FJMF. Body mass index and outcome in renal transplant recipients: a systematic review and meta-analysis. BMC Med. 2015;13(4S1 Suppl 1):111. doi:10.1186/s12916-015-0340-5 25963131 PMC4427990

[14] Hill CJ Courtney AE Cardwell CR, . Recipient obesity and outcomes after kidney transplantation: a systematic review and meta-analysis. Nephrol Dial Transplant. 2015;30(8):1403–1411. doi:10.1093/ndt/gfv214 26044837

[15] Sood A Hakim DN Hakim NS. Consequences of recipient obesity on postoperative outcomes in a renal transplant: a systematic review and meta-analysis. Exp Clin Transplant 2016 Apr;14(2):121–128. PMID: 27015529 27015529

[16] Yin S Wu L Huang Z Fan Y Lin T Song T. Nonlinear relationship between body mass index and clinical outcomes after kidney transplantation: A dose-response meta-analysis of 50 observational studies. Surgery. 2022;171(5):1396–1405. doi:10.1016/j.surg.2021.10.024 34838329

